# SAG-UPS regulates malignant transformation—from chronic inflammation to pro-tumorigenesis to liver cancer

**DOI:** 10.1038/cddis.2015.312

**Published:** 2015-10-22

**Authors:** S C Chang, J L Ding

**Affiliations:** 1 Department of Biological Sciences, National University of Singapore, Singapore 117543, Singapore; 2 The Ph.D. Program for translational Medicine, College for Medical Science and Technology, Taipei Medical University, 250 Wusing Street, Taipei 110, Taiwan

Apoptosis has been recognized as an important defense mechanism against infection.^1^ However, understanding of the checkpoint between apoptosis and immune response in tumorigenesis is lacking. Using *in vitro* and *ex vivo* approaches, Chang and Ding^2^ have recently shown that survival advantage to the macrophages during early infection is conferred by the protective potency of SAG (sensitive to apoptosis gene). SAG mediates antiapoptosis through ubiquitination of pro-apoptotic signaling factors. Studies with primary bone marrow-derived macrophage cells have demonstrated that SAG has a key regulatory role in balancing the ratio of pro- and antiapoptotic factors in the infection challenged macrophages, as indicated by the production of both pro- and anti-tumorigenic cytokines. SAG-ubiquitination of pro-apoptotic factors strategically supports macrophage survival, sustaining immune defense during early infection. This finding clearly established the power of SAG-UPS (ubiquitin-proteasome system) as a functional link between immune defense and apoptosis or immune-overactivation and tumorigenesis. Hence, SAG-UPS was proposed to be an efficient target for developing therapeutics against autoimmune diseases and cancers.

On the other hand, infection and chronic inflammation condition reduces intracellular antioxidant activities and increases the production of reactive oxygen species (ROS) that damages DNA, polyunsaturated fatty acids in lipids and amino acids in proteins.^3^ All of these contribute to the multi-stage malignant cellular transformation.^4^ As cancer often develops at the sites of chronic inflammation,^5^ it is perceived that chronic inflammation is the basis of tumor progression/establishment. This is supported by evidence that certain anti-inflammatory drugs reduce cancer risk.^6^ Low-grade persistent infection by hepatitis B virus can induce immune-overactivation leading to chronic inflammation. Therefore, it is crucial to elucidate the mechanisms that link chronic inflammation-induced immune responses with cancer biology. However, studies addressing the checkpoint between immune-overactivation and tumorigenesis are lacking. Hence, there is a great need to investigate the molecular mechanisms and key regulators involved in chronic infection-inflammation and cancer development. There are several lines of evidence hinting at a key switch between chronic inflammation and tumorigenesis. First, UPS-related proteins are often found to be overexpressed in cancer cells, for example, SAG.^7^ Second, SAG was reported to stimulate cancer growth via its dual-functions—ROS-scavenging antioxidant activity and E3 ubiquitin ligase activity.^7^ Third, SAG-dependent ubiquitination system has been shown to regulate immune defense and apoptosis in macrophage cells under infection condition.^2^ Furthermore, in this study authors have shown SAG to mediate inflammatory responses by manipulating proinflammatory cytokine production (e.g. IL-1*β*, IL-6 and TNF-*α*) in Mø. Based on the congruence of chronic inflammation, continuous expression of proinflammatory cytokines and recruitment of immune cells, where SAG-UPS feature prominently, Chang and Ding hypothesized that SAG-UPS switches the cells between immune-overactivation and pro-tumorigenesis. They explored novel apoptotic factors in chronic inflammation-associated hepatocellular carcinomas (HCC) from different stages of the disease, and discovered that SAG-UPS confers survival potency in early HCC, probably supporting disease progression and malignant transformation (Chang *et al.*^8^). Here, the authors showed that SAG-UPS degrades pro-apoptotic signaling factors, thus conferring antiapoptosis and uncontrolled cell survival strategy, which explains the promotion of liver cancer. The authors found that the levels of pro- (IL-1*β*, IL-6, TNF and IL-12p40) and anti- (IL-10) inflammatory cytokines in the primary HCCs correlate with chronic liver inflammation. They demonstrated that SAG is upregulated in the early stage carcinoma tissues and further showed that SAG-UPS perturbs the fine balance of the ratio of pro-apoptotic (SARM and Noxa) and antiapoptotic factors. Furthermore, *SAG*-overexpression significantly contributed to the production of pro-tumorigenic cytokines (IL-1*β*, IL-6 and TNF), but not the anti-inflammatory cytokine (IL-10) and anti-tumorigenic cytokine (IL-12p40). Based on these findings, Chang *et al.* postulate that SAG-UPS probably favors and exacerbates the vicious cycle of tumorigenic microenvironment for the progression of hepatoma (Figure 1). On the premise that SAG is most highly upregulated at the early stage of HCC, it was proposed that SAG-UPS is an efficient marker for early diagnosis as well as a potential target for development of therapeutics against chronic inflammation-associated hepatoma.

## Figures and Tables

**Figure 1:**
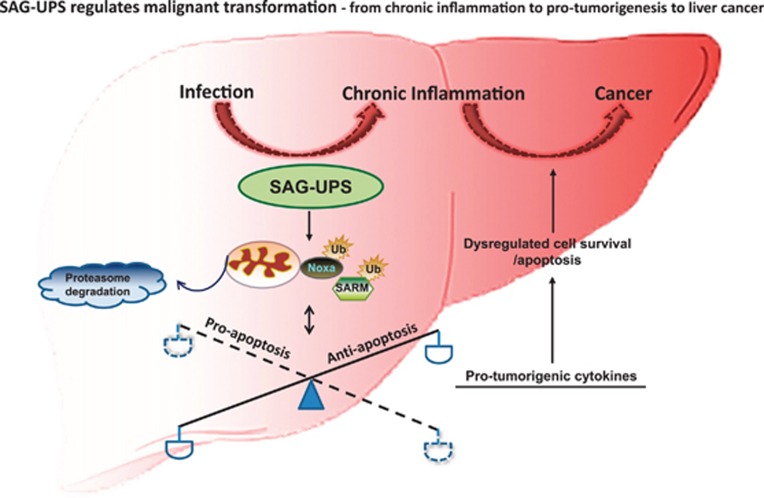
SAG-UPS regulates tumor progression—from hepatitis virus infection-induced chronic inflammation and pro-tumorigenesis to HCC. The left and right sections represent healthy condition and hepatocarcinogenesis, respectively. Chronic infection by hepatitis viruses (hepatitis B virus and hepatitis C virus) is a major risk factor for the initiation and development of HCC. During infection, upregulated SAG-UPS mediates autocrine signaling to attenuate pro-apoptotic Noxa and SARM by ubiquitination (Ub), leading to imbalanced anti-/pro-apoptotic factors. The activation of SAG also promotes pro-tumorigenic cytokines (e.g. IL-1*β*, TNF and IL-6), which, by paracrine signaling, exacerbates the tumor microenvironment and drives malignant transformation

## Abbreviations

HCC: hepatocellular carcinoma
SAG: sensitive to apoptosis gene
UPS: ubiquitin-proteasome system
